# Adherence to the Mediterranean Diet Is Associated with Health-Related Quality of Life and Anthropometric Measurements in University Professors

**DOI:** 10.3390/healthcare11131928

**Published:** 2023-07-03

**Authors:** María López-Olivares, Elisabet Fernández-Gómez, Miriam Mohatar-Barba, Trinidad Luque-Vara, Teresa Nestares, Marta López-Bueno, Carmen Enrique-Mirón

**Affiliations:** 1 Department of Nutrition and Food Science, Faculty of Health Sciences, Melilla Campus, University of Granada, 52001 Melilla, Spain; 2 Department of Nursing, Faculty of Health Sciences, Melilla Campus, University of Granada, C/Santander s/n, 52001 Melilla, Spain; 3 Instituto de Investigación Biosanitaria (ibs.GRANADA), 18014 Granada, Spain; 4 Department of Physiology, Faculty of Pharmacy, University of Granada, 18071 Granada, Spain; 5 Biomedical Research Centre (CIBM), Institute of Nutrition and Food Technology “José MataixVerdú” (INYTA), University of Granada, 18071 Granada, Spain; 6 Department of Inorganic Chemistry, HUM-613 Research Group, Faculty of Health Sciences, Melilla Campus, University of Granada, C/Santander s/n, 52001 Melilla, Spain

**Keywords:** PREDIMED, fat percentage, quality of life, education

## Abstract

The main objective of this study was to assess the relationship between Mediterranean diet (MD) adherence and health-related quality of life (HRQOL) according to the anthropometric measurements of teaching and research staff (TRS) at the University of Granada (UGR), Spain. This diagnostic, non-experimental, cross-sectional, and observational study was performed on university lecturers (65 women and 62 men) using a correlational descriptive methodology. The lecturers’ anthropometric measurements were taken, while MD adherence was determined using the PREvention with MEDiterranean diet (PREDIMED) questionnaire. The Short Form Health Survey (SF-36) was used for measuring HRQOL. Better results for body composition were associated with improvements in the physical and mental dimensions and MD adherence. Statistically significant differences were found between sexes, with men showing higher values for weight, height, waist circumference, BMI, waist/hip ratio (WHR), muscle mass, and systolic and diastolic pressure than women. Similarly, MD adherence was positively correlated with vitality (r = 0.233; *p* = 0.009), social functioning (r = 0.229; *p* = 0.008), and the mental component summary (r = 0.205; *p* = 0.021). The regression model determined that the mental component summary (β = 0.239, *p* = 0.041), diastolic pressure (PD) (β = −0.473, *p* < 0.000), fat percentage (FP) (β = −0.241, *p* = 0.004), and age (β = −0.231, *p* = 0.022) significantly predicted MD adherence. The results obtained in this study suggest that healthy dietary patterns such as the MD and an optimum body composition contribute to an improved HRQOL.

## 1. Introduction

Improving quality of life (QL) continues to be a challenge for research. The World Health Organization (WHO) defines quality of life as “an individual’s perception of their position in life in the context of the culture and value systems in which they live and in relation to their goals, expectations, standards and concerns” [1]. QL is both subjective and objective and is often classified by physical, material, social, and emotional dimensions, as well as development and activity [2]. In adults, it is also probable that QL is influenced by social aspects, such as life situations [3], financial dependence [4], age-related physical limitations [5,6], and lifestyle factors, including physical activity [7], diet, and nutrition [8,9].

The Mediterranean diet (MD) is considered to be one of the healthiest dietary models worldwide [10]. The Mediterranean diet has been researched extensively for its possible beneficial effects on various chronic diseases based on data obtained by the Seven Countries study in the 1960s [11]. In Spain, a large randomised nutritional intervention trial led by Martínez-González et al. (2015) [12] and called the PREDIMED (PREvención con DIeta MEDiterránea or PREvention with MEDiterranean diet) trial was initiated in 2002. Its main aim was to evaluate the MD’s efficacy in preventing cardiovascular diseases (CVDs). The incidences of other pathologies, including cancer (breast, colorectal, lung, and stomach) and diabetes, as well as global mortality, were also considered.

Several observational studies have associated adherence to the Mediterranean diet with improvements in BMI, muscle mass, fat mass, and blood pressure, as well as a greater longevity and quality of life [13,14,15,16,17,18,19,20,21]. However, adherence to the ‘Western’ dietary pattern based on the consumption of red meat, sugars, and fast food has a negative association [22]. The Molisani project [23], a cohort study based on an Italian population, and other cross-sectional studies performed in Spain [24,25] associated the traditional MD with a better health-related quality of life (HRQOL).

University teaching is important for training and educating professionals. However, the activity has personal risks associated with the pressures of academia and scientific lecturing, which, in turn, have a professional impact as they directly affect the educational process [26]. University lecturers are subject to excessive workloads, budgetary deficits, disputes with the deanery, and exhaustion from the pressure to publish [27,28,29,30,31,32]. Consequently, teaching staff are exposed to greater emotional challenges that may affect their quality of life and teaching ability [33,34,35].

In general, a better HRQOL has been associated with greater MD adherence; however, the improved health category dimensions (e.g., physical, emotional, and social) differ between these studies. Benefits were observed for the physical dimension in some studies [36,37], whereas only the mental dimension showed improvement in others [22,23,25]. Determining the reasons for these differences and the mechanisms involved remains a subject of investigation.

Evidence for the link between high MD adherence and HRQOL is important from a public health perspective, as it can reduce costs in the healthcare system and increase life expectancy. In this context, our general objective for this study was to assess the relationship between MD adherence, HRQOL, and the anthropomorphic measurements of the University of Granada’s (UGR) teaching and research staff (TRS).

## 2. Materials and Methods

### 2.1. Study Design and Procedure

Quantitative research was performed with a nonexperimental, ex post facto design since the study variables were not manipulated. This study was cross-sectional since the data were collected at a single timepoint. Furthermore, it was an exploratory and descriptive correlational study because we intended to describe the observed relationships among variables in a less-studied group.

The data were gathered between February 2019 and March 2020 at the UGR Melilla Campus. Participants completed the questionnaire in person after signing written informed consent forms. A nutritionist conducted the evaluation and the anthropometric measurements from participants, who fasted for 8 h or more and emptied their bladders. A qualified nurse took blood pressure measurements.

### 2.2. Participants

The sample comprised 127 university lecturers from the UGR Melilla Campus in the academic year 2019/2020, with an average age of 47.38 ± 11.37 years. The minimum age was 29 years and the maximum age was 67 years. The average ages were 49.4 ± 11.6 years for men and 45.2 ± 10.8 years for women. Table 1 includes the sociodemographic characteristics of the sample.

As shown in Table 1, men and women comprised 48.8% and 51.2% of those surveyed, respectively. Concerning the faculty, 40.2% belong to Education and Sport Sciences, 28.3% to Health Sciences, and 31.5% to Social and Legal Sciences. The majority of participants were from Melilla (63%), married (67.7%), and Christian by religion (61.6%), with professional stability (63%).

### 2.3. Instruments

A bioimpedance meter (inBody R20) (Biospace, Seoul, Republic of Korea) and mechanical telescopic measuring rod were used (SECA 222) (Seca gmbh, Hamburg, Germany) to determine anthropometric measurements (body weight (kg), height (cm), waist and hip circumference (cm)) and body composition (lean body mass (kg), percentage of body fat, and fat-free mass (kg)). BMI was calculated from weight and height and categorised as underweight (<18.5 kg/m^2^), normal weight (18.5–24.9 kg/m^2^), overweight (25–29.9 kg/m^2^), or obese (30.0 kg/m^2^) according to the criteria established by the OMS [38]. Blood pressure was determined using a Hylogi MD-H12 blood pressure monitor (Urion, Beijing, China).

To evaluate MD adherence, we used the 14-point questionnaire from the PREDIMED study [12], which was already validated for a similar cohort in Spain [39]. Higher scores in this questionnaire suggest greater adherence to foods characteristic of the MD. Scores ≥ 9 suggest high MD adherence, whereas scores < 9 indicate low MD adherence.

The Spanish language SF-36 Health Survey (version 2) was used for health-related variables. It comprises 36 items that represent health-related quality of life according to 8 subscales or dimensions: physical functioning (PF), physical role (PR), bodily pain (BP), general health (GH), vitality (VT), social functioning (SF), emotional role (ER), and mental health (MH). Furthermore, it can be used to determine the state of general health by calculating the total of two components from the following subscales: physical (PF + PR + BP + GH) and mental (ER + SF + MH + VT) [40]. The scores of each item are coded, added, and transformed on a scale from 0 to 100. The higher the score, the better the functioning.

### 2.4. Ethical Statement

This study was conducted following the directives established by the Declaration of Helsinki. All participants provided written informed consent. Approval was obtained from the Provincial Board of Education of Melilla (reference number 201802658) on 10 April 2018 and was presented by the vice dean of Internalization, Research and Transfer of the Faculty of Education and Humanities of the University of Granada.

### 2.5. Data Analysis

Statistical analyses were conducted with SPSS 26.0 software (IBM SPSS Statistics, Chicago, IL, USA). Visual and analytical methods were used to investigate the variables (Kolmogorov–Smirnov tests) and determine whether they had normal distributions. The descriptive values are presented as number (*n*), percentage (%), mean (x), standard deviation (SD), and median and interquartile range (IQR). The continuous variables were not normally distributed (non-parametric tests), and the Mann–Whitney U test was used to compare their averages. To calculate significant differences in prevalence, Pearson’s chi-square test was used. A non-parametric Spearman’s rank correlation analysis was performed to determine the relationships between numerical variables.

A multiple regression model and ordinary least squares estimate were used to evaluate the relationship between MD adherence, HRQOL, and sociodemographic and anthropometric measurements. The standardised and non-standardised regression coefficients (β) were calculated for all the regression models. Dispersion diagrams were generated to view the relationship between MD adherence scores and fat percentage, diastolic pressure, and the physical component summary. A *p*-value < 0.05 was considered statistically significant for all analyses.

## 3. Results

Table 2 shows the participants’ anthropometric characteristics and body composition according to sex. On average, the men weighed 81.9 kg and women weighed 64.5 kg. The women had a greater fat percentage (30.9%) than the men (27.5%).

As shown in Table 2, statistically significant differences were found between the sexes for weight, height, waist circumference, BMI, WHR (waist/hip ratio), muscle mass, and systolic and diastolic pressure. Men showed higher values for these variables while women showed higher fat percentages. Likewise, statistically significant differences were found between men and women in the BMI categories. The percentage of men who were overweight (53.3%) was higher than that of women, who were mostly normal weight (61.5%).

Considering the scores achieved in the PREDIMED questionnaire, 48.8% of participants had high MD adherence (scores ≥ 9), whereas 51.2% had low MD adherence (scores < 9) (Table 3). No statistically significant differences were observed between the sexes. However, the percentage of women with high MD adherence was slightly greater than that of men (50.2% of women compared with 46.8% of men). Table 3 shows the degree of compliance with MD recommendations in both subgroups and the average scores achieved: 8.29 ± 1.77 (total sample), 8.32 ± 1.75 (men), and 8.26 ± 1.80 (women). There was a statistically significant difference between men and women in the consumption of at least one daily portion of red meat, hamburger, sausage, or cold meat, with men having a greater consumption of these products.

Table 4 shows SF-36 scores for the eight dimensions and two component summaries according to sex. No significant differences in any of the dimensions or in the component summaries were observed.

Table 5 shows the correlation between the physical and mental component summaries, the SF-36 subscales, and the MD adherence questionnaire scores with anthropometric measurements and body composition.

In the case of men, negative correlations were found between WHR and mental health; BMI and physical role; fat percentage and physical functioning; emotional role and the physical component summary; and systolic pressure and physical functioning. In other words, WHR and BMI decreased as mental health and physical role increased, respectively. On the other hand, fat percentage decreased with the increase in physical functioning, emotional role, and the physical component summary. Similarly, as systolic pressure decreased, physical functioning increased.

In the case of women, WHR was negatively correlated with physical functioning and MD adherence; BMI with MD adherence; fat percentage with physical functioning; the physical component summary with MD adherence; and systolic pressure with MD adherence. Similarly, WHR decreased as physical functioning increased. A lower WHR and BMI were associated with higher scores for MD adherence. A lower fat percentage was inversely related to physical functioning, the physical component summary, and MD adherence. Finally, as systolic pressure decreased, the MD adherence score increased.

In the total sample, negative correlations were found between WHR and vitality; BMI and physical functioning, physical role, body pain, mental health, and MD adherence; fat percentage and physical functioning, physical role, the physical component summary, and MD adherence; systolic pressure and vitality; and diastolic pressure and MD adherence. As WHR and BMI decreased, there was an increase in vitality, physical functioning, physical role, bodily pain, the mental component summary, and the MD adherence score. Similarly, a lower fat percentage was associated with higher scores in physical functioning, physical role, the physical component summary, and MD adherence. Finally, lower systolic and diastolic pressure results were associated with a better vitality and MD adherence, respectively.

The association between MD adherence and SF-36 is shown in Table 6. Regarding sex, a relationship between social functioning and MD adherence was found in female participants (r = 0.238; *p* = 0.048). Simultaneously, social functioning increased with MD adherence. For males, a positive relationship was found between vitality (r = 0.407; *p* < 0.000), mental health (r = 0.297; *p* = 0.019), and the mental component summary (r = 0.336; *p* = 0.008). Similarly, better MD adherence scores were associated with better results for those variables. Finally, MD adherence in the total sample (r = 0.233; *p* = 0.009), vitality (r = 0.233; *p* = 0.009), social functioning (r = 0.229; *p* = 0.008), and mental component summary (r = 0.205; *p* = 0.021) were related, i.e., a better MD adherence score was associated with a better vitality, social functioning, and mental component summaries.

The results of the regression model, presented in Table 7, predict the effects of age, fat percentage, systolic pressure, diastolic pressure, the physical component summary, and the mental component summary on MD adherence. For the total sample, the mental component summary (β = 0.239, *p* = 0.041), DP (β = −0.473, *p* < 0.000), FP (β = −0.241, *p* = 0.004), and age (β = 0.231, *p* = 0.022) significantly predicted adherence to the Mediterranean diet.

## 4. Discussion

Several epidemiological studies have investigated MD and HRQOL factors, but not together. Nevertheless, our results indicate that MD adherence may be associated with an improved quality of life. We found statistically significant differences according to sex in BMI averages, the percentages of normal weight and overweight individuals, WHR, and fat percentage, with women showing better results. Some studies obtained similar results [41,42,43]. BMI is the most widely used method to determine overweight or obesity prevalence. However, in recent years, this indicator has been deemed inadequate for measuring fat distribution [44,45]. Various alternatives to BMI consider fatty tissue concentrations, e.g., X-ray absorptiometry, magnetic resonance, computed tomography, body fat percentage by bioelectric impedance (BIA), WHR, and other more complex techniques [45,46]. In addition to BMI, we used advanced bioimpedance (BIA) techniques and WHR in our study.

Previous studies suggested that obesity occurs when the body fat percentage exceeds 25% in men and 35% in women [47,48]. In our study, men exceeded these limits more than women. Body fat percentage is strongly associated with risks of chronic diseases such as hypertension, dyslipidaemia, diabetes mellitus, and heart disease [49]. On the other hand, some studies have suggested that WHR combined with BMI may be a better indicator for evaluating the relationship between obesity and health [50]. According to the WHO (2008) [51], a low risk of CVD is associated with WHRs below 0.90 and 0.85 in men and women, respectively. For the men in this study, the mean BMI of 26.2 kg/m^2^ and WHR of 0.89 were not within the established limits. By contrast, the mean values for women were within the normal limits, at 23.9 kg/m^2^ for BMI and 0.78 cm for WHR. Similar results were found in a similar population of lecturers [52].

Considering the results obtained from the MD adherence questionnaire, 51.2% of the participants had low adherence compared with 48.8% that had good adherence. No statistically significant differences were obtained between the sexes; however, it should be highlighted that there were more men (53.2%) with lower MD adherence than women (49.2%). Research on the dietary habits of university lecturers is very scarce, while university students are usually shown to have low MD adherence in the literature [53,54,55]. According to sex, there were statistically significant differences in the consumption of red meat, hamburger, sausage, or cold meat. Men ingested a greater amount of this food group compared to women. In this regard, reducing red meat intake is necessary to lower the risk of chronic diseases. On the other hand, some studies have shown that consuming less than the recommended amount of red meat is associated with depressive or anxiety disorders [56,57].

This study did not find significant differences in quality of life perception between the sexes. Conversely, Louzado (2021) [58] asserted that sex is a determining factor for adult quality of life. Based on our dimension analysis, we observed that vitality affects men more than women, which suggests that females may have greater energy for life activities.

In the total sample, vitality, physical functioning, physical role, bodily pain, the mental component summary, and the MD adherence score increased when WHR and BMI decreased. Likewise, a lower fat percentage was associated with higher scores for physical functioning, physical role, the physical component summary, and MD adherence. Finally, lower systolic and diastolic pressure values were associated with a better vitality and MD adherence, respectively. A study performed by Zaragoza-Martí et al. [59] shared similarities with our study in that subjects with good MD adherence had lower BMIs and body weight percentages. Research conducted by Mikkola [60], Stephenson [61], and Burgos-Postigo et al. [62] supports our results, which demonstrate that individuals with excess fat have a worse perception of quality of life than those of normal weight.

In our sample of university professionals, vitality, social functioning, and the mental component summary were positively correlated with the total sample’s MD adherence. Similarly, in studies conducted in Spain [36] and Greece [22], a significant association was observed between MD adherence, all domains of physical health, and most mental health domains. Other investigations have reached similar conclusions, finding significant differences mainly in the mental domain [21,24].

Finally, the mental component summary, diastolic pressure, fat percentage, and age significantly predicted MD adherence, consistent with other studies [58,63,64]. Subjects with good MD adherence had lower body fat percentages, lower blood pressure, and better emotional states.

The results of this study must be interpreted considering some limitations. Firstly, more studies are required to clarify these associations in a general sample of university lecturers. Secondly, this cross-sectional study only represents the group’s current situation. Therefore, establishing any cause–effect relationship is not possible. Thirdly, our study depended on subjectively informed variables. For this reason, future research may benefit from additional objective indicators.

Our study may be one of the first to consider TRS, a less-studied population in the scientific literature. Therefore, performing similar research on a national level with a representative sample of university lecturers would benefit future research.

## 5. Conclusions

In our study, we found that adherence to the Mediterranean dietary pattern is largely associated with the mental component summary of university lecturers at the University of Granada on the Melilla campus, as well as with fat percentage and blood pressure. Similarly, the better the body composition of the TRS, the better the SF-36 questionnaire results obtained in the physical and mental dimensions.

The results presented in this study provide opportunities for future research into whether MD adherence is not only correlated with a better quality of life but also causal. If the relationship is causal, MD adherence may become an approach to improving the population’s emotional and physical experiences, which would involve benefits beyond well-being.

## Figures and Tables

**Table 1:** Sociodemographic variables studied (results in frequencies and percentages; *n* = 127).

Variables		Frequency (%)
Sex	Male	62 (48.8)
	Female	65 (51.2)
Faculty	Education and Sport Sciences	51 (40.2)
	Health Sciences	36 (28.3)
	Social and Legal Sciences	40 (31.5)
Origin	Melilla	80 (63.0)
	Rest of Spain	47 (37.0)
Relationship status	Single	26 (20.5)
	Married	86 (67.7)
	In a couple	10 (7.9)
	Separated	3 (2.3)
	Divorced	1 (0.8)
	Widowed	1 (0.8)
Professional stability	Yes	80 (63.0)
	No	47 (37.0)
Religion	Christian	98 (61.6)
	Muslim	5 (3.1)
	Non-believer	14 (8.8)
	Other	10 (26.5)

Professional stability: it is the type of employment contract that the respondent had.

**Table 2:** Participants’ anthropometric measurements, body composition, and BMI category according to sex.

Variables	Men (*n* = 62)		Women (*n* = 65)		*p*-Value
	x ± SD	Median (IQR)	x ± SD	Median (IQR)	
Weight (kg)	81.9 ± 11.0	82.1 (16.3)	64.5 ± 12.4	62.40 (13.4)	<0.000 ^a^
Height (cm)	174.4 ± 7.3	173.0 (8.2)	164.8 ± 5.6	165.00 (9.0)	<0.000 ^a^
Waist circumference (cm)	92.7 ± 11.4	91.5 (14.2)	80.0 ± 10.4	78.0 (16.0)	<0.000 ^a^
Hip circumference (cm)	103.4 ± 8.2	102.0 (11.0)	101.9 ± 8.7	101.0 (13.0)	0.173
BMI (kg/m^2^)	26.2 ± 4.2	25.45 (3.9)	23.9 ± 4.1	22.60 (6.1)	0.002
WHR	0.89 ± 0.1	0.88 (0.1)	0.78 ± 0.1	0.77 (0.1)	<0.000 ^a^
Muscle mass (kg)	33.1 ± 5.2	33.50 (6.9)	24.0 ± 3.3	23.90 (3.7)	<0.000 ^a^
Fat mass (kg)	21.9 ± 8.7	19.35 (12.7)	20.6 ± 8.3	18.00 (12.6)	0.280
Fat percentage (%)	27.5 ± 8.7	25.35 (12.7)	30.9 ± 7.9	29.50 (13.5)	0.020 ^b^
Blood pressure (mm Hg)					
Systolic pressure	122.8 ± 14.0	120.00 (20.0)	110.3 ± 11.9	110.00 (20.0)	<0.000 ^a^
Diastolic pressure	74.4 ± 11.1	72.50 (16.2)	67.5 ± 8.9	65.00 (10.0)	<0.000 ^a^
	Frequency (%)		Frequency (%)		*p*-value
BMI category (kg/m^2^)					
Low weight (<18.5)	0		1 (1.5)		0.282
Normal weight (18.5–24.9)	22 (35.5)		40 (61.5)		<0.000 ^a^
Overweight (25.0–29.9)	33 (53.3)		18 (27.7)		<0.000 ^a^
Obesity (>30)	7 (11.2)		6 (9.3)		0.374

x: mean; SD: standard deviation; BMI: body mass index; WHR: waist/hip ratio; IQR: interquartile range. Statistically significant differences between the groups evaluated were analysed using the Mann–Whitney U and chi-square tests. *p*-value: ^a^ <0.001 ^b^ < 0.05

**Table 3:** Compliance with MD recommendations and MD adherence according to sex. Frequencies and percentages of affirmative answers.

MD Recommendations	Men (*n* = 62)	Women (*n* = 65)	*p*-Value
	Frequency (%)	Frequency (%)	
Use of olive oil as main fat	61 (98.4)	62 (95.4)	0.335
Consumption of ≥4 spoonfuls of olive oil per day	57 (91.9)	53 (81.5)	0.087
Consumption of ≥2 portions (100 g) of vegetables per day	15 (24.2)	22 (33.8)	0.233
Consumption of ≥3 portions (pieces) of fruit a day	19 (30.6)	25 (38.5)	0.357
Consumption of <1 portion (100–150 g) of red meat, hamburger, sausage, or cold meat per day	42 (67.7) ^c^	55 (84.6) ^c^	0.026
Consumption of <1 portion (12 g) of butter, margarine, or cream per day	51 (82.3)	52 (80.0)	0.746
Consumption of <1 portion of carbonated and/or sugary drink per day	48 (77.4)	53 (81.5)	0.567
Consumption of ≥1 portion of wine per week	16 (25.8)	11 (16.9)	0.223
Consumption of ≥3 portions (150 g) of legumes per week	17 (27.4)	14 (21.5)	0.442
Consumption of ≥3 portions (100–150 g) of fish per week	34 (54.8)	28 (43.1)	0.187
Consumption of <3 portions of non-homemade cakes/pastries per week	40 (64.5)	44 (67.7)	0.706
Consumption of ≥3 portions (30 g) of pulses per week	39 (62.9)	37 (56.9)	0.494
Preference for chicken or turkey meat over pork, hamburger, or sausage (100–150 g)	44 (71.0)	47 (72.3)	0.868
Consumption of ≥2 portions of vegetables, pasta, rice, or other dishes cooked/seasoned with vegetable-based sauce per week	32 (51.6)	34 (52.3)	0.987
	x ± SD	x ± SD	*p*-value
PREDIMED score (0 to 14 points)	8.32 ± 1.7	8.26 ± 1.8	0.589
	Frequency (%)	Frequency (%)	*p*-value
MD adherence			
High (PREDIMED score ≥ 9)	29 (46.8)	33 (50.8)	0.653
Low (PREDIMED score < 9)	33 (53.2)	32 (49.2)	

Statistically significant differences between the groups were analysed using chi- square tests. *p*-value: ^c^ < 0.05.

**Table 4:** Participants’ physical component summaries (PCSs), mental component summaries (MCSs), and SF-36 subscales.

Dimensions of SF-36	Men (*n* = 62)	Women (*n* = 65)	*p*-Value
	Mean ± SD	Mean ± SD	
Physical Functioning	93.1 ± 12.6	90.8 ± 15.6	0.091
Physical Role	91.4 ± 14.3	92.2 ± 14.3	0.830
Bodily Pain	69.0 ± 20.2	68.3 ± 22.3	0.953
General Health	68.3 ± 15.5	70.0 ± 17.9	0.454
Vitality	56.5 ± 14.2	51.7 ± 17.0	0.210
Social Functioning	86.5 ± 17.6	84.8 ± 20.6	0.798
Emotional Role	87.0 ± 18.9	89.7 ± 16.0	0.606
Mental Health	49.3 ± 10.6	48.7 ± 12.6	0.609
Physical Component Summary	74.8 ± 7.4	74.7 ± 8.2	0.835
Mental Component Summary	55.1 ± 7.6	54.3 ± 8.6	0.646

In all cases, *p*-value > 0.050. Statistical significance was calculated with Mann–Whitney U tests for ordinary data analyses of the participants according to sex.

**Table 5:** Correlation between physical and mental component summaries, SF-36 subscales, MD adherence questionnaire, anthropometric measurements, and body composition.

	PF	PR	BP	GH	VT	SF	ER	MH	PCS	MCS	MD
Male											
WHR	−0.248	−0.006	−0.068	0.217	0.225	0.230	0.045	−0.285 ^b^	−0.001	0.228	0.049
BMI	−0.245	−0.263 ^b^	−0.144	0.089	0.113	0.002	−0.113	0.244	−0.139	0.111	−0.027
FP (%)	−0.298 ^b^	−0.212	−0.109	−0.124	−0.220	−0.056	−0.282 ^b^	−0.099	−0.260 ^b^	−0.177	−0.118
SP	−0.302 ^b^	−0.186	0.059	−0.097	0.153	−0.034	−0.220	0.167	−0.200	0.067	0.006
DP	−0.223	−0.028	0.022	−0.048	0.082	−0.032	−0.121	0.024	−0.101	0.007	−0.167
Female											
WHR	−0.400 ^a^	−0.138	−0.028	−0.106	0.165	−0.064	0.099	0.033	−0.172	0.121	−0.312 ^b^
BMI	−0.225	−0.213	−0.218	−0.117	−0.076	−0.030	−0.039	−0.083	−0.238	−0.114	−0.335 ^a^
FP (%)	−0.284 ^b^	−0.181	−0.198	−0.118	−0.043	−0.015	0.054	−0.082	−0.259 ^b^	−0.079	−0.352 ^a^
SP	−0.109	0.015	0.003	−0.041	0.173	−0.023	0.021	0.009	−0.032	0.071	−0.302 ^b^
DP	−0.171	−0.002	−0.093	0.008	0.131	0.042	0.101	0.018	−0.041	0.103	−0.232
Total											
WHR	−0.161	−0.099	−0.066	−0.002	−0.239 ^a^	0.070	0.029	0.172	−0.081	0.174	−0.112
BMI	−0.183 ^b^	−0.243 ^a^	−0.180 ^b^	−0.032	0.060	−0.003	−0.080	0.103	−0.192 ^b^	0.017	−0.179 ^b^
FP (%)	−0.305 ^a^	−0.182 ^b^	−0.151	−0.102	−0.140	−0.29	−0.105	−0.107	−0.247 ^a^	−0.138	−0.228 ^b^
SP	−0.118	−0.081	0.023	−0.87	−0.177 ^b^	−0.027	−0.117	0.074	−0.104	0.057	−0.159
DP	−0.149	−0.019	−0.37	−0.49	0.123	−0.02	−0.051	0.030	−0.081	0.41	−0.194 ^b^

PF (physical functioning); PR (physical role); BP (bodily pain); GH (general health); VT (vitality); SF (social functioning); ER (emotional role); MH (mental health); PCS (physical component summary); MCS (mental component summary); WHR (waist/hip ratio); BMI (body mass index); FP (fat percentage); SP (systolic pressure); DP (diastolic pressure); MD (Mediterranean diet). Spearman’s rank correlation coefficient test was performed. *p*-value: ^a^ <0.01; ^b^ <0.05.

**Table 6:** Association between MD adherence and SF-36.

SF-36	Mediterranean Diet Adherence					
	Total Sample		Male		Female	
	R	*p*-Value	r	*p*-Value	r	*p*-Value
PF	0.143	0.108	0.102	0.430	0.181	0.149
PR	0.113	0.206	0.064	0.619	0.159	0.206
BP	0.057	0.521	−0.039	0.765	0.141	0.263
GH	0.142	0.110	0.086	0.506	0.183	0.144
VT	0.233	0.009 ^b^	0.407	0.000 ^a^	0.091	0.471
SF	0.229	0.008 ^b^	0.217	0.090	0.238	0.048 ^c^
ER	0.097	0.278	0.159	0.218	0.026	0.837
MH	0.149	0.095	0.297	0.019 ^c^	0.022	0.864
PCS	0.145	0.105	0.013	0.922	0.214	0.085
MCS	0.205	0.021 ^c^	0.336	0.008 ^b^	0.085	0.500

PF (physical functioning); PR (physical role); BP (bodily pain); GH (general health); VT (vitality); SF (social functioning); ER (emotional role); MH (mental health); PCS (physical component summary); MCS (mental component summary). The Spearman’s rank correlation coefficient test was performed. *p*-value: ^a^ <0.001; ^b^ <0.01; ^c^ <0.05.

**Table 7:** Multiple linear regression models for evaluating the relationship between MD adherence score and variables in the total sample (*n* = 127).

Variable	Mediterranean Diet Adherence		
	B	SE	β
(Constant)	8.532 ^a^	2.010	
Age	0.036 ^c^	0.015	0.231
Fat percentage	−0.051 ^b^	0.018	−0.241
Systolic pressure	0.021	0.014	0.167
Diastolic pressure	−0.076 ^a^	0.018	−0.473
Physical component summary	0.025	0.017	0.243
Mental component summary	0.013 ^c^	0.019	0.239

*p*-value: ^a^ <0.001; ^b^ <0.01; ^c^ <0.05.

## References

[1] Organización Mundial de la Salud (OMS) (1995). Introducción, Administración, Puntuación y Versión Genérica de la Evaluación. Programa de Salud Mental, Organización Mundial de la Salud. https://apps.who.int/iris/handle/10665/203851.

[2] Felce D., Perry J. (1995). Calidad de vida: Su definición y medición. Res. Desarro. Deshabilitar.

[3] Lee E., Cha S., Kim G.M. (2021). Factors Affecting Health-Related Quality of Life in Multimorbidity. Healthcare.

[4] Wan S.E., Siwar C., Zaidi M.A.S., Abdul-Kadir H. (2019). Health related quality of life (HRQOL) among low socioeconomic population in Malaysia. BMC Public Health.

[5] Ferreira L.N., Pais S., Ilchuk K., Santos M. (2022). Envejecimiento, calidad de vida relacionada con la salud y actividad física—Evidencia basada en el EQ-5D-5L. Envejec. Int..

[6] Krawczyk-Suszek M., Kleinrok A. (2022). Health-Related Quality of Life (HRQoL) of People over 65 Years of Age. Int. J. Environ. Res. Public Health.

[7] Zurita-Ortega F., Salvador-Pérez F., Knox E., Gámiz-Sánchez V.M., Chacón-Cuberos R., Rodríguez-Fernández S., Muros-Molina J.J. (2018). Actividad física y calidad de vida relacionada con la salud de los escolares: Análisis de ecuaciones estructurales. Anal. Psicol..

[8] Vajdi M., Farhangi M.A. (2020). A systematic review of the association between dietary patterns and health-related quality of life. Health Qual. Life Outcomes.

[9] Catherine M., Milte M.G., Thorpe D., Kylie S.A. (2015). Associations of diet quality with health-related quality of life in older Australian men and women. Exp. Gerontol..

[10] Morris L., Bhatnagar D. (2016). La dieta mediterránea. Actual. Opinión Lipidol..

[11] Keys A., Menotti A., Karvonen M.J., Aravanis C., Blackburn H., Buzina R., Djordjevic B.S., Dontas A.S., Findanza F., Keys M. (1986). La dieta y la tasa de mortalidad a los 15 años en el Estudio de siete países. D. Am. Epidemiol..

[12] Martínez-González M.A., Salas-Salvadó J., Estruch R., Corella D., Fitó M., Ros E., Predimed Investigators (2015). Mediterranean Diet for Primary Prevention of Cardiovascular Disease. Prog. Cardiovasc. Dis..

[13] Estruch R., Ros E., Salas-Salvadó J., Covas M.I., Corella D., Arós F., Gómez-Gracia E., Ruiz-Gutiérrez V., Fiol M., Lapetra J. (2013). Primary prevention of cardiovascular disease with a Mediterranean diet. N. Engl. J. Med..

[14] Estruch R., Ros E., Salas-Salvadó J., Covas M.I., Corella D., Arós F., Gómez-Gracia E., Ruiz-Gutiérrez V., Fiol M., Lapetra J. (2018). PRedimed study primary prevention of cardiovascular disease with a mediterranean diet supplemented with extra-virgin olive oil or nuts. N. Engl. J. Med..

[15] Schröder H., Marrugat K., Vila J., Covas M., Elosua R. (2014). Adherence to the Traditional Mediterranean Diet Is Inversely Associated with Body Mass Index and Obesity in a Spanish Population. J. Nutr..

[16] Agarwal A., Ioannidis J.P.A. (2019). Predimed trial of Mediterranean diet: Retracted, republished, still trusted?. BMJ.

[17] Salucci S., Bartoletti-Stella A., Bavelloni A., Aramini B., Blalock W.L., Fabbri F., Vannini I., Sambri V., Stella F., Faenza I. (2022). Extra Virgin Olive Oil (EVOO), a Mediterranean Diet Component, in the Management of Muscle Mass and Function Preservation. Nutrients.

[18] Tian H., Qiu R., Jing L., Chen Z., Chen G., Chen Y. (2017). Alternate Mediterranean diet score is positively associated with skeletal muscle mass index in middle-aged adults. Br. J. Nutr..

[19] Schroder H., Mendez M., Ribas-Barba L., Covas M.I., Serra-Majem L. (2011). Dieta mediterránea y circunferencia de la cintura en una muestra nacional representativa de jóvenes españoles. Rev. Int. Obes. Pediátrica.

[20] Dominguez L.J., Di Bella G., Veronese N., Barbagallo M. (2021). Impact of Mediterranean Diet on Chronic Non-Communicable Diseases and Longevity. Nutrients.

[21] Galilea-Zabalza I., Buil-Cosiales P., Salas-Salvadó J., Toledo E., Ortega-Azorín C., Díez-Espino J., Vázquez-Ruiz Z., Zomeño M.D., Vioque J., Martínez J.A. (2018). Dieta mediterránea y calidad de vida: Análisis transversal de referencia del ensayo PREDIMED-PLUS. PLoS ONE.

[22] Ruano C., Henriquez P., Martínez-González M.V., Bes-Rastrollo M., Ruiz-Canela M., Sánchez-Villegas A. (2013). Proyecto de patrones dietéticos derivados empíricamente y calidad de vida relacionada con la salud en el proyecto SUN. PLoS ONE.

[23] Bonaccio M., Di Castelnuovo A., Bonanni A., Costanzo S., De Lucia F., Pounis G., Benedetta M., Gaetano G., Lacoviello L., Moli-sani investigators (2013). La adherencia a una dieta mediterránea se asocia con una mejor calidad de vida relacionada con la salud: Un posible papel del alto contenido de antioxidantes en la dieta. BMJ.

[24] Cadarso-Suárez A., Dopico-Calvo X., Iglesias-Soler E., Cadarso-Suárez C.M., Gude-Sampedro F. (2017). Calidad de vida relacionada con la salud y su relación con la adherencia a la dieta mediterránea y la actividad física en universitarios de Galicia. Nutr. Clínica Y Dietética Hosp..

[25] Sánchez-Aguadero N., Alonso-Dominguez R., Garcia-Ortiz L., Agudo-Conde C., Rodriguez-Martin C., de Cabo-Las A., Sanchez-Salgado B., Ramos R., Maderuelo-Fernandez J.A., Gomez-Marcos M.A. (2016). Dieta y actividad física en personas con riesgo cardiovascular intermedio y su relación con la calidad de vida relacionada con la salud: Resultados del estudio MARK. Result. Calid. Vida Salud.

[26] Madrid S.D.P.C., Moreno M.P., Beltrán C.A. (2020). Riesgos psicosociales en docentes universitarios. Recimundo.

[27] Osifila G., Aladetan T. (2020). Workload and lecturers’ job satisfaction in Adekunle Ajasin University, Akungba-Akoko, Ondo State, Nigeria. J. Educ. Learn..

[28] Collado P.A., Soria C.B., Canafoglia E., Collado S.A. (2016). Health and working conditions of high school and university teachers in Mendoza: Between commitment and emotional distress. Salud Colect..

[29] Pace F., D’Urso G., Zappulla C., Pace U. (2021). The relation between workload and personal well-being among university professors. Curr. Psychol..

[30] David I.C., Quintão S. (2012). Burnout in teachers: Its relationship with personality, coping strategies and life satisfaction. Acta Med. Port..

[31] Tijdink J.K., Vergouwen A.C.M., Smulders Y.M. (2013). Publication pressure and burn out among Dutch medical professors: A nationwide survey. PLoS ONE.

[32] Sestili C., Scalingi S., Cianfanelli S., Mannocci A., Del Cimmuto A., De Sio S., Chiarini M., Di Muzio M., Villari P., De Giusti M. (2018). Reliability and Use of Copenhagen Burnout Inventory in Italian Sample of University Professors. Int. J. Environ. Res. Public Health.

[33] Alves P.C., Oliveira A.F., Paro H.B.M.D.S. (2019). Calidad de vida y agotamiento entre los miembros de la facultad: Cuánto importa el campo del conocimiento?. PLoS ONE.

[34] Rodríguez-Mantilla J.M., Fernández-Díaz M.J. (2017). The effect of interpersonal relationships on burnout syndrome in Secondary Education teachers. Psicothema.

[35] Skaalvik E.M., Skaalvik S. (2017). Dimensions of teacher burnout: Relations with potential stressors at school. Soc. Psychol. Educ..

[36] Pérez-Tasigchana R.F., León-Muñoz L.M., López-García E., Banegas J.R., Rodríguez-Artalejo F., Guallar-Castillón P. (2016). Dieta Mediterránea y Calidad de Vida Relacionada con la Salud en Dos Cohortes de Adultos Mayores Comunitarios. PLoS ONE.

[37] Henríquez-Sánchez P., Ruano C., de Irala J., Ruiz-Canela M., Martínez-González M.A., Sánchez-Villegas A. (2012). Adherencia a la dieta mediterránea y calidad de vida en el Proyecto SUN. Eur. J. Clin. Nutr..

[38] Organización Mundial de la Salud (OMS) (2021). Obesidad y Sobrepeso. https://www.who.int/es/news-room/fact-sheets/detail/obesity-and-overweight.

[39] Guillem P., Wang Y., Guillem J., Guadalupe V., Saiz C. (2017). Estilos de vida, adherencia a la dieta mediterránea, características antropométricas en un colectivo de universitarios de ciencias de la salud. Rev. Esp. Nutr. Comunitaria.

[40] Rodriguez-Romero B., Pita-Fernández S., Pertega S., Chouza-Insua M. (2013). Calidad de vida relacionada con la salud en trabajadoras del sector pesquero usando el cuestionario SF-36. Gac. Sanit..

[41] Hall J.A., Ochoa Martínez P.Y., Sáenz-López Buñuel P., Monreal Ortíz L.R. (2009). Estudio comparativo del nivel de actividad física, estado nutricio y obesidad abdominal en profesores de educación física de la Universidad Autónoma de Sinaloa y la Universidad de Huelva. Nuevas Tend. Educ. Fís. Deporte Recreación.

[42] Paredes A.F., Pancca D.C., Ccopa S.A., Saico C.R.Y., Vanegas Y.M.P. (2021). Actividad física, estrés y su relación con el índice de masa corporal en docentes universitarios en pandemia. Rev. Investig. Comun. Desarro..

[43] Rodríguez-guzmán L., Díaz-cisneros F.J., Rodríguez-guzmán E. (2016). Sobrepeso y obesidad en profesores. Anales de la Facultad de Medicina.

[44] Zaslavsky O., Rillamas-Sun E., LaCroix A.Z., Woods N.F., Tinker L.F., Zisberg A., Shadmi E., Cochrane B., Edward B.J., Kritchevsky S. (2016). Association Between Anthropometric Measures and Long-Term Survival in Frail Older Women: Observations from the Women’s Health Initiative Study. J. Am. Geriatr. Soc..

[45] Lee G., Choi S., Park S.M. (2021). Association of waist circumference with muscle and fat mass in adults with a normal body mass index. Nutr. Res. Pract..

[46] Wollner M., Paulo B.B., Roncally S.C., Jurandir N., Edil L.S. (2017). Accuracy of the WHO’s body mass index cut-off points to measure gender- and age-specific obesity in middle-aged adults living in the city of Rio de Janeiro, Brazil. J. Public Health.

[47] De Lorenzo A., Deurenberg P., Pietrantuono M., Di Daniele N., Cervelli V., Andreoli A. (2003). How fat is obese?. Acta Diabetol..

[48] Organización Mundial de la Salud (OMS) (1995). Physical Status: The Use and Interpretation of Anthropometry: Report of a WHO Expert Committee. https://apps.who.int/iris/handle/10665/37003.

[49] Goswami B., Reang T., Sarkar S., Sengupta S., Bhattacharjee B. (2020). Role of body visceral fat in hypertension and dyslipidemia among the diabetic and nondiabetic ethnic population of Tripura-A comparative study. J. Family Med. Prim Care.

[50] Zhang F.L., Ren J.-X., Zhang P., Jin H., Qu Y., Yu Y., Guo Z.-N., Yang Y. (2021). Strong Association of Waist Circumference (WC), Body Mass Index (BMI), Waist-to-Height Ratio (WHtR), and Waist-to-Hip Ratio (WHR) with Diabetes: A Population-Based Cross-Sectional Study in Jilin Province, China. J. Diabetes Res..

[51] Organización Mundial de la Salud (OMS) (2008). Waist Circumference and Waist-Hip Ratio. http://apps.who.int/iris/bitstream/handle/10665/44583/9789241501491_eng.pdf?sequence=1.

[52] Molano-Tobar N.J., Ordoñez-Fernández M.Y., Molano-Tobar D.X. (2017). Cambios antropométricos y asociación del nivel de actividad física en docentes universitarios. Rev. Cienc. Cuid..

[53] Karam J., Bibiloni M.D.M., Serhan M., Tur J.A. (2021). Adherence to Mediterranean Diet among Lebanese University Students. Nutrients.

[54] López-Moreno M., Garcés-Rimón M., Miguel M., Iglesias López M.T. (2021). Adherence to Mediterranean Diet, Alcohol Consumption and Emotional Eating in Spanish University Students. Nutrients.

[55] Redondo del Río M.P., Mateo Silleras B., Carreño Enciso L., Marugán de Miguelsanz J.M., Fernández McPhee M., Camina Martín M.A. (2016). Ingesta dietética y adherencia a la dieta mediterránea en un grupo de estudiantes universitarios en función de la práctica deportiva. Nutr. Hosp..

[56] Jacka F.N., Pasco J.A., Williams L.J., Mann N., Hodge A., Brazionis L., Berk M. (2012). Red meat consumption and mood and anxiety disorders. Psychother. Psychosom..

[57] Opie R.S., O’Neil A., Itsiopoulos C., Jacka F.N. (2015). The impact of whole-of-diet interventions on depression and anxiety: A systematic review of randomised controlled trials. Public Health Nutr..

[58] Louzado J.A., Lopes M., Galvão M., Moraes V., Mistro S., Souto D., Arruda D., Oliveira K., Nicolaevna C., Honorato V.C. (2021). Gender Differences in the Quality of Life of Formal Workers. Int. J. Environ. Res. Public Health.

[59] MJ C.M., JA H.S. (2015). Adherence to the mediterranean diet and its relation to nutritional status in older people. Nutr. Hosp..

[60] Mikkola T.M., Kautiainen H., von Bonsdorff M.B., Salonen M.K., Wasenius N., Kajantie E., Eriksson J.G. (2020). Body composition and changes in health-related quality of life in older age: A 10-year follow-up of the Helsinki Birth Cohort Study. Qual. Life Res..

[61] Stephenson J., Smith C.M., Kearns B., Haywood A., Bissell P. (2021). The association between obesity and quality of life: A retrospective analysis of a large-scale population-based cohort study. BMC Public Health.

[62] Burgos-Postigo B., Duarte A., Fernández A., García O. (2019). Relación entre la Calidad de Vida y el Índice de Masa Corporal (IMC) en una Muestra de Trabajadores. Kronos.

[63] Azorín M., Martínez M., Sánchez A.B., De la Ossa M., Hernández I., Tello G.M., Párraga I. (2018). Adherencia a la dieta mediterránea en pacientes hipertensos en Atención Primaria. Rev. Clin. Med. Fam..

[64] Rodríguez-González M., Tárraga-Marcos M.L., Madrona-Marcos F., Sadek I.M., Celada-Roldan C., Tárraga-López P.J. (2019). Efectos de la dieta mediterránea sobre los factores de riesgo cardiovascular. J. Negat. No Posit. Results.

